# Epigenetic targeting of PGBD5-dependent DNA damage in SMARCB1-deficient sarcomas

**DOI:** 10.1172/JCI179282

**Published:** 2025-08-12

**Authors:** Yaniv Kazansky, Helen S. Mueller, Daniel Cameron, Phillip Demarest, Nadia Zaffaroni, Noemi Arrighetti, Valentina Zuco, Prabhjot S. Mundi, Yasumichi Kuwahara, Romel Somwar, Rui Qu, Andrea Califano, Elisa de Stanchina, Filemon S. Dela Cruz, Andrew L. Kung, Mrinal M. Gounder, Alex Kentsis

**Affiliations:** 1 Molecular Pharmacology Program, Sloan Kettering Institute and; 2 Tow Center for Developmental Oncology, Department of Pediatrics, Memorial Sloan Kettering Cancer Center, New York, New York, USA.; 3 Molecular Pharmacology Unit, Department of Experimental Oncology, Fondazione IRCCS Istituto Nazionale dei Tumori di Milano, Milan, Italy.; 4 Department of Systems Biology, Columbia University Irving Medical Center, New York, New York, USA.; 5 Department of Biochemistry and Molecular Biology, Kyoto Prefectural University of Medicine, Japan.; 6 Department of Pathology and; 7 Antitumor Assessment Core, Memorial Sloan Kettering Cancer Center, New York, New York, USA.; 8 Herbert Irving Comprehensive Cancer Center and; 9 Departments of Biochemistry and Molecular Biophysics, Biomedical Informatics, and Medicine, Vagelos College of Physicians and Surgeons, Columbia University, New York, New York, USA.; 10 Chan Zuckerberg Biohub New York, New York, New York, USA.; 11 Department of Pediatrics and; 12 Department of Medicine, Memorial Sloan Kettering Cancer Center, New York, New York, USA.; 13 Departments of Pediatrics, Pharmacology, and Physiology & Biophysics, Weill Medical College of Cornell University, New York, New York, USA.

**Keywords:** Cell biology, Oncology, DNA repair, Drug therapy, Epigenetics

## Abstract

Despite the potential of targeted epigenetic therapies, most cancers do not respond to current epigenetic drugs. The polycomb repressive complex EZH2 inhibitor tazemetostat was recently approved for the treatment of *SMARCB1*-deficient epithelioid sarcomas, based on the functional antagonism between PRC2 and SMARCB1. Through the analysis of tumors of patients treated with tazemetostat, we recently defined key principles of their response and resistance to EZH2 epigenetic therapy. Here, using transcriptomic inference from *SMARCB1*-deficient tumor cells, we nominate the DNA damage repair kinase ATR as a target for rational EZH2 combination epigenetic therapy. We showed that EZH2 inhibition promotes DNA damage in epithelioid and rhabdoid tumor cells, at least in part via its induction of piggyBac transposable element derived 5 (PGBD5). We leveraged this collateral synthetic lethal dependency to target PGBD5-dependent DNA damage by inhibition of ATR, but not CHK1, using the ATR inhibitor elimusertib. Consequently, combined EZH2 and ATR inhibition improved therapeutic responses in diverse patient-derived epithelioid and rhabdoid tumors in vivo. This advances a combination epigenetic therapy based on EZH2-PGBD5 synthetic lethal dependency suitable for immediate translation to clinical trials for patients.

## Abstract 2

Rational combination of EZH2 and ATR inhibition improves therapeutic response in SMARCB1-deficient tumors, through epigenetic induction of transposase-derived PGBD5.

## Introduction

Targeted epigenetic therapies offer potential improvements over conventional cytotoxic chemotherapies through superior clinical efficacy and reduced toxicity. In cancers caused by genetic mutations of transcriptional and epigenetic regulators, specific inhibition of epigenetic effectors can directly block dysregulated gene expression by leveraging cancer-specific dependencies. One promising example of this therapeutic approach is in cancers caused by mutation of the chromatin remodeling SWI/SNF/BAF (Brg/Brahma-associated factors) complex, a ubiquitous epigenetic regulator that is mutated in at least 20% of human cancers (1). In particular, the highly lethal solid tumors caused by loss of the core BAF subunit *SMARCB1* (2–4), which include malignant rhabdoid tumors and epithelioid sarcomas, are known to be dependent on the methyltransferase EZH2, a component of the polycomb repressive complex 2 (PRC2). This dependency is thought to result from epigenetic antagonism between BAF and PRC2, in which normal BAF activity evicts PRC2 from tumor suppressor loci (5, 6). In a recent clinical trial, this dependency was targeted using the EZH2 methyltransferase inhibitor tazemetostat, leading to its FDA approval (7).

Yet despite the promise of this therapeutic approach, EZH2 inhibition as a monotherapy exhibited efficacy in only a subset of patients, with most patient tumors either having primary resistance or acquiring resistance after treatment (7, 8). Thus, there is a critical need to develop improved epigenetic combination therapies that can be achieved, at least in part, through improved understanding of the effects of EZH2 inhibition in epithelioid and rhabdoid tumors.

We recently leveraged functional genomics of epithelioid and rhabdoid tumors to elucidate the mechanisms of resistance to EZH2 inhibition, including frequent disruption of the RB1/E2F cell cycle control axis causing tazemetostat resistance in patients. This discovery led to the identification of a combination therapy approach using treatment with the AURKB inhibitor barasertib to overcome *RB1*-mediated tazemetostat resistance in vitro and improve therapeutic responses in preclinical epithelioid and rhabdoid tumor xenografts in vivo (8). This approach bypasses primary and acquired RB1/E2F-mediated checkpoint defects to maintain tazemetostat-induced reprogramming of oncogenic gene expression. However, RB1/E2F pathway alterations were observed in less than half of the epithelioid and rhabdoid tumors of patients. Thus, further investigation into understanding cellular tazemetostat -dependent effects is needed to define rational combination therapies. This is necessary to achieve effective epigenetic therapy for patients with an intact RB1/E2F axis, including those with primary or acquired tazemetostat resistance.

Epithelioid and rhabdoid tumors occur due to characteristic biallelic deletions and inactivating mutations of *SMARCB1*, either as a result of germline rhabdoid tumor predisposition syndrome or due to somatic mutations (2, 9, 10). For rhabdoid tumors, differences in the types of mutations (e.g., large versus small chromosomal deletions or missense and nonsense mutations of *SMARCB1*) correspond with distinct molecular subtypes and clinical features (11). Genome sequencing studies have identified distinct sequence-specific mutational features in rhabdoid tumors, including sequence-specific deletions of *SMARCB1* and recurrent mutations of other genes, associated, at least in part, with the mutagenic activity of the domesticated transposase-derived piggybac transposable element derived 5 (PGBD5) (9).

*PGBD5* is among the most evolutionarily conserved, domesticated, transposase-derived genes in vertebrates and can mediate sequence-specific DNA rearrangements dependent on its putative nuclease activity (9, 12). In particular, Pgbd5 promotes sequence-specific somatic mutagenesis and tumor development in mouse models of medulloblastoma, one of the most common childhood brain tumors (13). PGBD5-expressing cells, including rhabdoid tumor cells, require a distinct form of nonhomologous end-joining DNA repair, leading to their hypersensitivity to inhibition of DNA repair signaling, specifically ATR inhibition (14).

We have now found that tazemetostat-mediated inhibition of EZH2 can increase PGBD5 expression in epithelioid and rhabdoid tumor cells, potentiating PGBD5-dependent DNA damage and conferring synergy with the ATR-selective DNA repair inhibitor elimusertib. Accordingly, combined treatment of patient-derived rhabdoid and epithelioid tumors engrafted into immunodeficient mice, including from patients with multiply relapsed metastatic disease, demonstrates synergistic activity in vivo.

## Results

**Figure 1:**
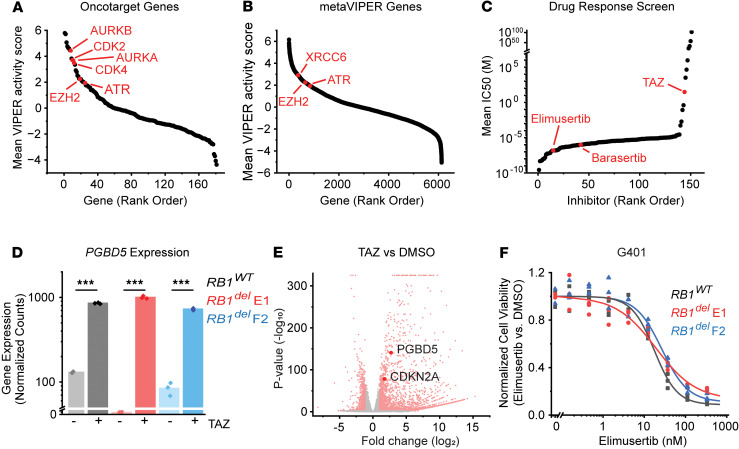
ATR inhibition is a therapeutic target and shows activity in tazemetostat-resistant rhabdoid tumor cells. (**A** and **B**) metaVIPER analysis of rhabdoid tumor transcriptomes for proteins within the OncoTarget protein set (**A**) and the complete metaVIPER protein set (**B**). (**C**) Inhibitors rank ordered by IC_50_, data from Lee, et al. (**D**) DESeq2-normalized read counts of cells treated with 10 μM tazemetostat (TAZ) versus equivalent volume of DMSO for 11 days. *n* = 3 biological replicates per condition. ***P < 0.001; *P* = 1.2 × 10^–7^, 1.3 × 10^–6^, 1.6 × 10^–6^, and for *RB1^WT^*, *RB1^del^* E1, and *RB1^del^* F2, respectively, by 2-sided Student’s *t* test. (**E**) Volcano plot of previously published RNA-Seq data from cells treated with 10 μM TAZ vs. equivalent volume of DMSO for 11 days. *n* = 3 replicates per condition. Dots in red indicate genes with log_2_(expression fold-change) > ±1 and *P* < 0.01. (**F**) G401 cells treated with elimusertib for 4 days (experiment was repeated 3 times; representative is shown here).

To define potential therapeutic targets for tazemetostat combination therapy in *SMARCB1*-deficient tumors, we leveraged a recently developed method for transcriptomic inference of protein activity, termed metaVIPER (virtual inference of protein activity by enriched regulon), which was recently applied to gene expression profiles of 68 patients’ rhabdoid tumors collected as part of the Therapeutically Applicable Research to Generate Effective Treatments (TARGET) initiative (15–17). To prioritize actionable targets, we focused the analysis on the previously published OncoTarget annotation of 180 putative master regulator (MR) proteins with high-affinity pharmacologic inhibitors available (17). We ordered these druggable candidates by their mean activity scores in this tumor cohort (Figure 1A and Supplemental Table 1; supplemental material available online with this article; https://doi.org/10.1172/JCI179282DS1). Together, these analyses identified potentially targetable proteins based on their transcriptional activity in these tumors. As expected, EZH2 was among the most activated MR proteins in rhabdoid tumors, as was AURKB, the target of our previously identified tazemetostat and barasertib combination therapy (Figure 1A). This analysis also prioritized CDK4, CDK2, and AURKA (Figure 1A), consistent with the previously established activity of cell cycle checkpoints downstream of G1/S in rhabdoid tumors (8), validating this ranking approach.

In addition to these known dependencies, this analysis also identified the nonhomologous end-joining DNA repair structural factor XRCC6 (Ku70), and the DNA repair kinase ATR among the most activated MR genes of all 6,000 MR proteins in metaVIPER (Figure 1B and Supplemental Table 2), both of which were shown in previous studies to be required for the survival of cells expressing active PGBD5 (14). Indeed, XRCC6 ranked higher in this analysis than the well-validated target EZH2 (Figure 1B).

To ascertain the robustness of this prediction, we next analyzed an independent data set from Lee et al. (18), which we used to assess the responses of sarcoma cell lines to a panel of 151 pharmacologic inhibitors, including G401 and A204 rhabdoid tumor and VA-ES-BJ epithelioid sarcoma cell lines. We observed that the ATR-selective kinase inhibitor elimusertib (BAY-1895344; mean IC_50_ = 146 nM) was among the most active drugs against epithelioid and rhabdoid tumor cells, and notably, more active than both barasertib (mean IC_50_ = 951 nM) and tazemetostat (mean IC_50_ > 100 mM) (Figure 1C and Supplemental Table 3). Our previous work and that of others have shown that the minimum tazemetostat treatment duration to observe cell viability effects in rhabdoid tumor cell lines is 7 days; therefore, the high mean IC_50_ for tazemetostat is likely a result of the shorter 72-hour treatment time used in this experiment. Thus, *SMARCB1*-deficient epithelioid and rhabdoid tumors are highly sensitive to ATR inhibition (14).

Since modulation of both gene expression by tazemetostat and DNA repair by ATR inhibitor elimusertib appears to have prominent activity in rhabdoid tumor cells, we inquired whether the combination of both drugs could achieve improved antitumor effects. We next observed that in addition to the expected upregulation of polycomb gene sets, which we described previously (8), EZH2 inhibition in G401 rhabdoid tumor cells via 11-day tazemetostat treatment also caused a significant increase in the expression of *PGBD5*, a key mediator of ATR sensitivity in rhabdoid tumors (mean increase of 6.6-fold and *P* = 1.2 × 10^–7^ by Student’s *t* test) (Figure 1D). This upregulation was more pronounced and more significant than upregulation of the validated EZH2 target *CDKN2A* (8) (Figure 1E). Given both this substantial upregulation of *PGBD5* by tazemetostat and the fundamental functions that *PGBD5* plays as a developmental mutator and an inducer of DNA damage in rhabdoid tumorigenesis (9, 12, 13), we pursued the therapeutic implications of tazemetostat-mediated *PGBD5* upregulation in tumor cells.

Using CRISPR gene editing, we previously generated isogenic *RB1* WT and *RB1^del^* mutant G401 cells and confirmed correct biallelic *RB1* inactivating mutations and consequent loss of RB1 protein expression, using the *AAVS1* safe harbor as a negative control (8). We found that loss of *RB1*, as well as other defects in the RB1/E2F axis, cause tazemetostat resistance (8). Consistent with the independence of this tazemetostat resistance mechanism from ATR inhibitor susceptibility, two independent G401 *RB1^del^* mutant cell lines also exhibited significant tazemetostat-mediated induction of *PGBD5* expression (mean fold-increase of 333 and 8.6; *P* = 1.3 × 10^–6^ and 1.6 × 10^–6^, by *t* test, for E1 and F2 clones, respectively) (Figure 1D). Additionally, both *RB1^WT^* and *RB1^del^* mutant G401 cells exhibited nanomolar susceptibility to elimusertib (EC_50_ = 18 ± 1.6 nM, 19 ± 3.8 nM, and 27 ± 3.2 nM for *RB1^WT^*, *RB1^del^* E1, and F2 clones, respectively; Figure 1F).

**Figure 2:**
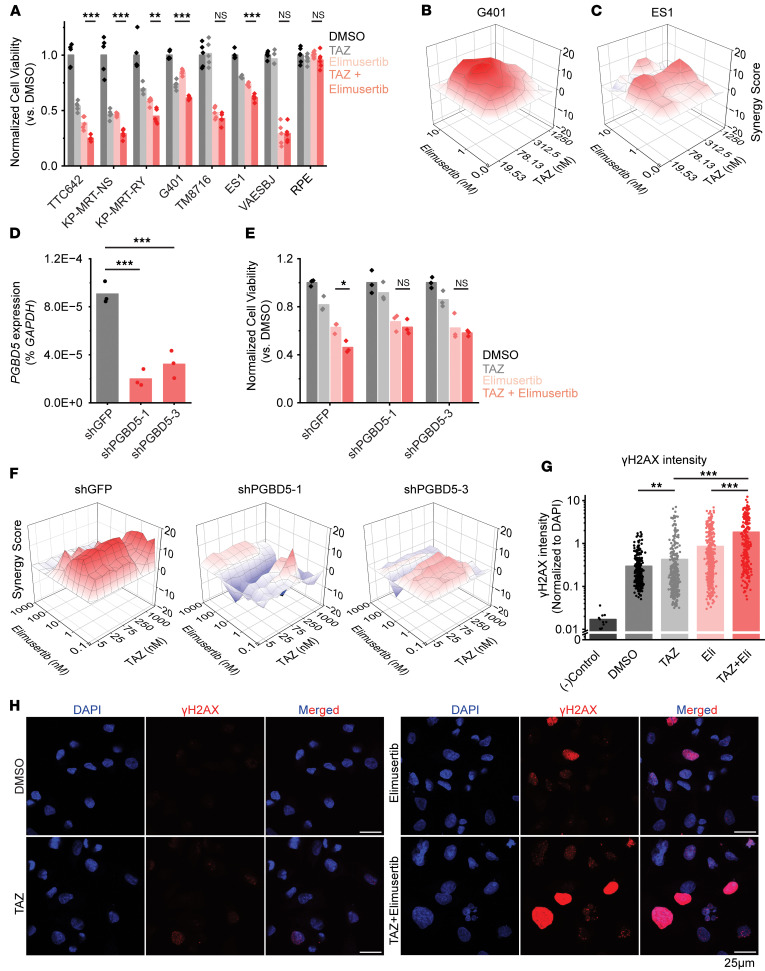
Combination EZH2 and ATR inhibition improves response in vitro. (**A**) Panel of rhabdoid (TTC642, KP-MRT-NS, KP-MRT-RY, G401, TM8716), epithelioid (ES1, VAESBJ) and nonsarcoma (RPE) cell lines ordered by decreasing response to TAZ monotherapy. Cells were treated with the indicated regimens at 200 nM TAZ and 8 nM elimusertib for 11 days. *P* = 0.001, 3.0 × 10^–5^, 0.0033, 2.6 × 10^–4^, 0.14, 6.1 × 10^–5^ for TTC642, KP-MRT-NS, KP-MRT-RY, G401, TM8716, and ES1, respectively, by 2-sided Student’s *t* test. All comparisons refer to combination versus elimusertib conditions, except for G401 cells, for which the comparison is for combination versus TAZ. *n* = 5 replicates per condition. (**B** and **C**) Synergy plots for combination treatment with TAZ and elimusertib for (**B**) G401 and (**C**) ES1 cells. Cells were treated for 9 days and analyzed for synergy using the ZIP model. (**D**) RT-qPCR showing *PGBD5* expression versus *GAPDH* in G401 cells with the indicated shRNA. P values were 2.8 × 10^–4^ and 8.0 × 10^–4^ for shGFP versus shPGBD5-1 and shPGBD5-3, respectively, by 2-sided Student’s *t* test with Bonferroni correction. (**E**) G401 cells with indicated shRNA were treated for 9 days with the indicated drug regimen (50 nM TAZ and 0.5 nM elimusertib). *P* = 0.01 for shGFP elimusertib versus combination by 2-sided Student’s *t* test. *n* = 3 replicates per condition. (**F**) ZIP synergy plots for the indicated cell line, treated for 9 days. (**G**) Quantification of γH2AX fluorescence relative to DAPI fluorescence. G401 cells were treated with 500 nM TAZ for 7 days ± 100 nM elimusertib (Eli) added on day 5. *P* = 0.0088 for DMSO versus TAZ; *P* = 1.2 × 10^–4^ for elimusertib versus combination; and TAZ versus combination by 2-sided permutation test with Benjamini-Hochberg (FDR) correction. *n* = 332 nuclei for DMSO, 404 for TAZ, 400 for elimusertib, 257 for combination. (**H**) Representative images of cells from (**G**). *P < 0.05, **P < 0.01, ***P < 0.001.

Elimusertib is currently undergoing clinical trials in patients with refractory or relapsed solid tumors, including patients with PGBD5-expressing tumors such as rhabdoid and epithelioid sarcoma (ClinicalTrials.gov NCT05071209). Therefore, we investigated the activity of combination treatment with tazemetostat and elimusertib, reasoning that tazemetostat-induced upregulation of *PGBD5* expression may potentiate the antitumor effects of ATR inhibition. We selected an elimusertib dose below its monotherapy IC_50_ (for G401 cells) to visualize any additive effects upon combination with tazemetostat. We observed greater antitumor effects with the combination of tazemetostat and elimusertib than with either drug alone in diverse rhabdoid and epithelioid sarcoma cell lines, including tazemetostat-resistant ES1 cells (mean decrease in normalized cell viability of 1.56-, 1.59-, 1.28-, 1.19-, and 1.19-fold compared with most effective monotherapy for TTC642, KP-MRT-NS, KP-MRT-RY, G401, and ES1, respectively; *P* = 1.0 × 10^-3^, 3.0 × 10^–5^, 3.3×10^-3^, 2.6 × 10^–4^, and 6.1 × 10^–5^, by *t* test, respectively; TM8716 cells showed a similar trend but did not reach the level of significance [*P* = 0.14 by *t* test]) (Figure 2A). In contrast, the combination did not impair proliferation in immortalized retinal pigment epithelial (RPE) cells (Figure 2A). Strikingly, the combination of tazemetostat and elimusertib was highly synergistic in G401 rhabdoid and ES1 epithelioid sarcomas cells (zero interaction potency [ZIP] synergy = 3.4 and 2, respectively) (Figure 2, B and C).

We next sought to confirm whether this synergistic effect is dependent on PGBD5 expression. To test this prediction, we engineered shRNA-mediated depletion of *PGBD5* in G401 cells using lentiviral transduction of two independent *PGBD5*-specific shRNAs (shPGBD5), as compared with a control GFP-targeting shRNA (shGFP). We confirmed that shPGBD5-expressing cells were significantly depleted of PGBD5 as compared with control shGFP cells (mean fold-depletion = 4.5 and 2.8; *P* = 2.8 × 10^–4^ and 8.0 × 10^–4^, by Bonferroni-adjusted *t* test, respectively) (Figure 2D). As predicted, control tumor cells transduced with shGFP vectors expressing endogenous levels of PGBD5 demonstrated significant reduction in cell viability when treated with the combination (1.4-fold vs. elimusertib, *P* = 0.013, by *t* test) (Figure 2E). In contrast, PGBD5-depleted shPGBD5-transduced cells showed no such reduction, which was also evident across all drug doses tested in the combination synergy analysis (Figure 2F).

If the improved antitumor activity of tazemetostat and elimusertib is due to the induction of PGBD5-dependent DNA damage, then this treatment combination should be associated with the induction of DNA damage repair signaling. To test this prediction, we used confocal immunofluorescence microscopy to quantify γH2AX as a specific marker of DNA damage (19). In agreement with prior studies (14), vehicle-treated G401 cells showed measurable γH2AX staining associated with baseline *PGBD5* expression (Figure 2, G and H). Consistent with tazemetostat-mediated induction of *PGBD5* expression (Figure 1D), we found that tazemetostat treatment alone significantly increased nuclear γH2AX (mean normalized intensity = 0.42 vs. 0.29 for tazemetostat and DMSO, respectively; *P* = 0.0088, Benjamini-Hochberg adjusted permutation test) (Figure 2, G and H). Additionally, the combination of tazemetostat and elimusertib induced a significant increase in γH2AX as compared with either drug alone (mean normalized intensity = 1.8 for the combination vs. 0.42 and 0.85 for tazemetostat and elimusertib, respectively; *P* = 1.2 × 10^–4^, Benjamini-Hochberg adjusted permutation test, for combination versus either elimusertib or tazemetostat monotherapy) (Figure 2, G and H).

**Figure 3:**
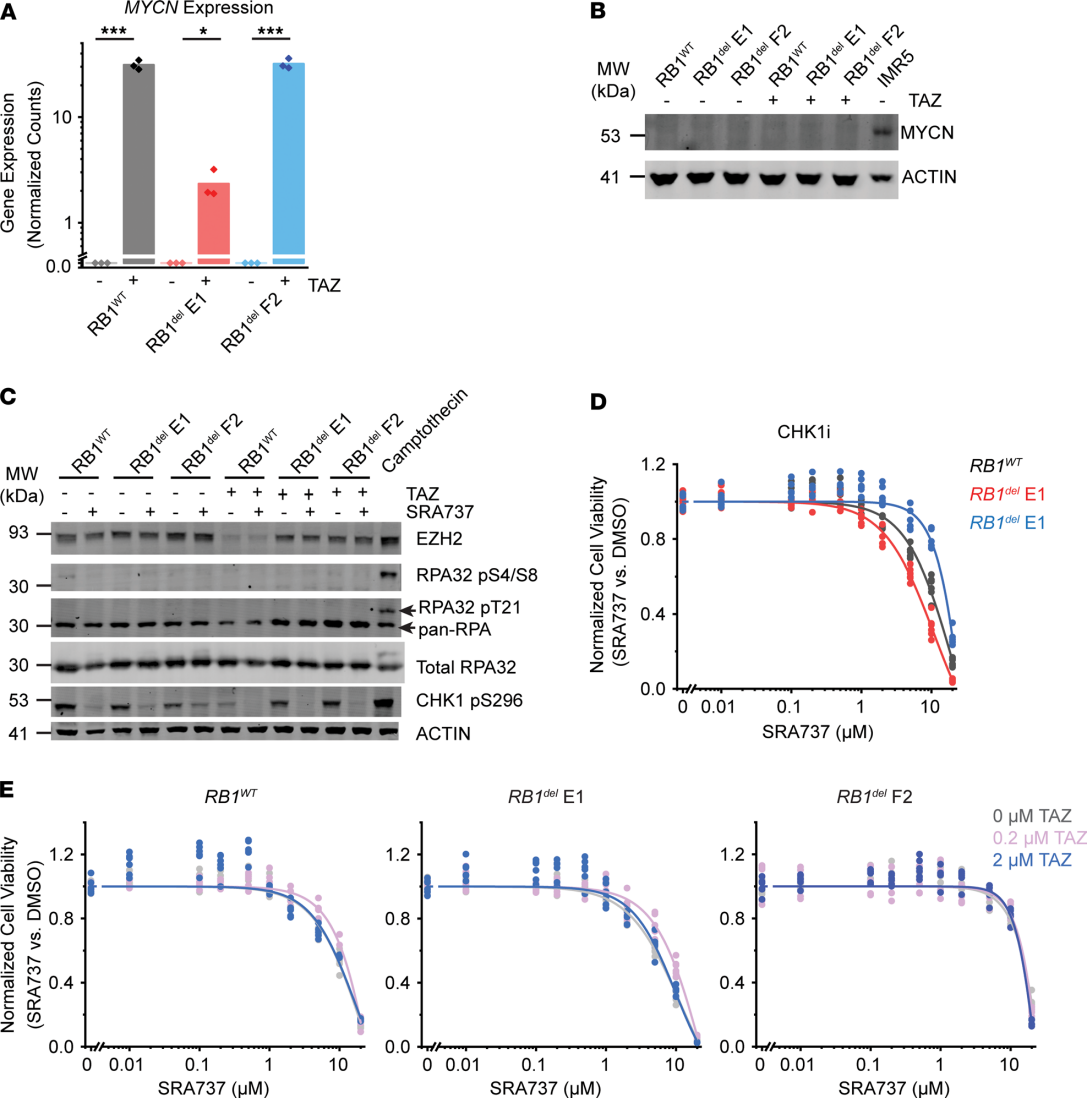
CHK1 inhibition does not induce replication stress or synergize with tazemetostat. (**A**) DESeq2-normalized read counts of cells treated with 10 μM TAZ versus equivalent volume of DMSO for 11 days. *n* = 3 biological replicates per condition. *P* = 0.0035, 0.032, and 0.0041 by 2-sided Student’s *t* test for DMSO versus TAZ-treated *RB1^WT^*, *RB1^del^* E1, and *RB1^del^* F2 cells, respectively. (**B**) Cells treated with 10 μM TAZ or DMSO for 11 days do not express MYCN protein. *MYCN*-amplified neuroblastoma cell line IMR5 was used as a positive control for MYCN expression. (**C**) Western blot assaying replication stress as measured by RPA32 phosphorylation at S4/8 and T21. Camptothecin treatment (1.5 μM) for 2 hours was used as a positive control for replication stress. Total RPA32 was used to control for RPA32 protein levels, and autophosphorylation of CHK1 at S296 was used to confirm CHK1 inhibition. Cells were pretreated with 10 μM TAZ or DMSO for 9 days. Cells were then split and additionally treated with SRA737 (3 μM) or equivalent volume of DMSO for 2 days. (**D**) Dose-response curves of G401 cells treated with the CHK1 inhibitor SRA737 for 9 days. (**E**) Dose-response curves of the indicated G401 cells treated with SRA737 for 9 days in combination with the indicated (0, 0.2, or 2.0 μM) TAZ concentration. Experiments in **D** and **E** were repeated 3 times; representative experiments are shown. *P < 0.05, ***P < 0.001.

Recently, EZH2 suppression was shown to induce replication stress through upregulation of MYCN expression in T acute lymphoblastic leukemia cells, which, in turn, sensitized cells to inhibition of CHK1 (19), a downstream mediator of ATR signaling (20). To test this possibility, we measured *MYCN* expression in G401 rhabdoid tumor cells, which was significantly increased upon tazemetostat treatment (Figure 3A; *P* = 0.0035, 0.032, and 0.0041, by *t* test, for DMSO- versus tazemetostat-treated *RB1^WT^*, *RB1^del^* E1, and *RB1^del^* F2 cells, respectively). However, this induction of *MYCN* gene expression was not associated with the accumulation of MYCN protein, as measured by Western blotting with *MYCN*-amplified IMR5 neuroblastoma cells as a positive control (Figure 3B). Consistent with this, EZH2 inhibition, either alone or in combination with CHK1 inhibition, did not induce apparent replication stress, as measured by RPA phosphorylation, using cells treated with the DNA topoisomerase inhibitor camptothecin as a positive control for replication stress and RPA phosphorylation. This was despite the effective inhibition of CHK1 autophosphorylation by the CHK1-selective SRA737 inhibitor (Figure 3C). Concordantly, the CHK1 inhibitor SRA737 showed poor activity against G401 cells, regardless of *RB1* status (Figure 3D), and sensitivity did not improve with the addition of 0.2 or 2.0 μM tazemetostat (Figure 3E). Thus, rhabdoid tumor cells exhibit a specific dependency on ATR-dependent but CHK1-independent DNA damage repair signaling.

**Figure 4:**
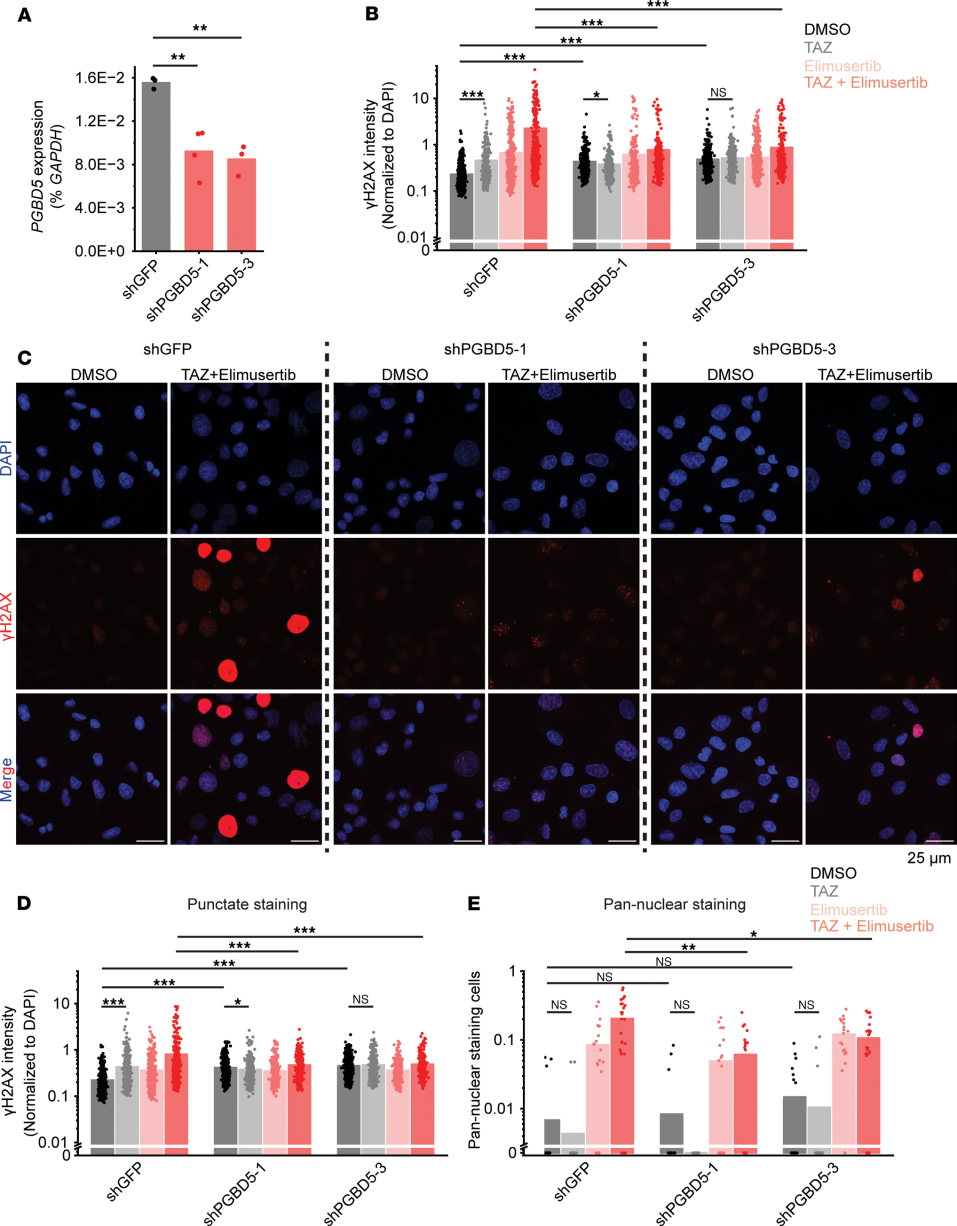
Tazemetostat-induced DNA damage is PGBD5 dependent. (**A**) RT-qPCR showing *PGBD5* expression versus *GAPDH* in G401 cells with the indicated shRNA. *P* = 0.0042 and 0.0033 for shGFP versus shPGBD5-1 and shPGBD5-3, respectively, by 2-sided Student’s *t* test with Bonferroni correction. (**B**) Quantification of γH2AX fluorescence relative to DAPI in all nuclei. *P* = 1.8 × 10^–4^ for both combination-treated shPGBD5-1 versus shGFP and shPGBD5-3 versus shGFP by 2-sided permutation test. *P* = 1.8 × 10^–4^, 0.011, and 0.26 for TAZ versus DMSO for shGFP, shPGBD5-1, and shPGBD5-3, respectively. *P* = 1.8 × 10^–4^ for DMSO-treated shPGBD5-1 versus shGFP and shPGBD5-3 versus shGFP. Cells were treated with the indicated regimen (500 mM TAZ for 7 days, and 100 nM elimusertib, added on day 5). (**C**) Representative images of cells quantified in (**B**). (**D**) Quantification of γH2AX fluorescence relative to DAPI in nuclei with punctate γH2AX staining. *P* = 1.7 × 10^–4^ for combination-treated shPGBD5-1 versus shGFP and shPGBD5-3 versus shGFP by 2-sided permutation test. *P* = 1.7 × 10^–4^, 0.047, and 0.29 for TAZ versus DMSO for shGFP, shPGBD5-1, and shPGBD5-3, respectively. *P* = 1.7 × 10^–4^ for DMSO-treated shPGBD5-1 versus shGFP and shPGBD5-3 versus shGFP. For **B** and **C**, n = 539, 497, and 703, respectively, for shGFP, shPGBD5-1, and shPGBD5-3 cells is 539, 497, and 703 for DMSO; *n* = 321, 390, and 418 for TAZ; *n* = 491, 354, and 554 for elimusertib; and *n* = 418, 277, and 317 for combination, respectively (**E**). Proportion of nuclei with pan-nuclear γH2AX staining per field. Each dot represents 1 field. *P* = 0.002 and 0.025 for combination-treated shPGBD5-1 versus shGFP and shPGBD5-3 versus shGFP, respectively, by 2-sided permutation test. *P* = 0.65, 0.30, and 0.65 for TAZ versus DMSO for shGFP, shPGBD5-1, and shPGBD5-3, respectively. *P* = 0.84 and 0.30 for DMSO-treated shPGBD5-1 versus shGFP and shPGBD5-3 versus shGFP, respectively. For shGFP cells, *n* = 22 fields for DMSO, TAZ, and elimusertib; *n* = 28 for combination. For shPGBD5-1, *n* = 22 for DMSO, TAZ, and elimusertib; *n* = 20 for combination. For shPGBD5-3, *n* = is 22 for DMSO, TAZ, and combination; and *n* = 20 for elimusertib. *P* values are for permutation tests adjusted with Benjamini-Hochberg correction. *P < 0.05, **P < 0.01, ***P < 0.001.

Tazemetostat-mediated induction of *PGBD5* and DNA damage would indicate that this form of DNA damage should be dependent on *PGBD5* expression. To test this prediction, we engineered additional G401 cells with shRNA-mediated depletion of *PGBD5* using lentiviral transduction of two independent shPGBD5 proteins, as compared with a control shGFP. We again confirmed that shPGBD5-expressing cells were significantly depleted of *PGBD5* as compared with control shGFP cells (mean fold-depletion = 1.7- and 1.9-fold; *P* = 0.0042 and 0.0033, by Bonferroni-adjusted *t* test, respectively) (Figure 4A). We note that these cells had a lower level of *PGBD5* depletion compared with the cells used in Figure 2, D–F, because G401 cells are difficult to culture long term with a high degree of *PGBD5* depletion.

We then measured DNA damage using quantitative confocal immunofluorescence microscopy of γH2AX, and found that, although the combination of tazemetostat and elimusertib induced DNA damage in shPGBD5 cells, this effect was significantly reduced compared with control shGFP cells (mean fold-reduction = 2.9- and 2.6-fold; *P* = 1.8 × 10^–4^, by Benjamini-Hochberg adjusted permutation test, for both combination-treated shGFP versus shPGBD5-1 and shPGBD5-3) (Figure 4, B and C). This PGBD5-dependent reduction in DNA damage was observed both when examining all nuclei with γH2AX staining (Figure 4B) and only nuclei with punctate γH2AX staining, corresponding to more localized DNA damage (mean fold-reduction = 1.7- and 1.6-fold; *P* = 1.7 × 10^–4^, by permutation test, for shGFP versus shPGBD5-1 and shPGBD5-3, respectively) (Figure 4D), as opposed to pan-nuclear γH2AX staining due to genome-wide unrepaired DNA damage and cellular apoptosis (mean fold-reduction = 3.2- and 1.8-fold; *P* = 0.002 and 0.025, by permutation test, for shGFP versus shPGBD5-1 and shPGBD5-3, respectively) (Figure 4E). Thus, in addition to its requirement for tazemetostat and elimusertib synergy, PGBD5, at least in part is necessary for tazemetostat-mediated induction of DNA damage and its potentiation by the combination with elimusertib.

**Figure 5:**
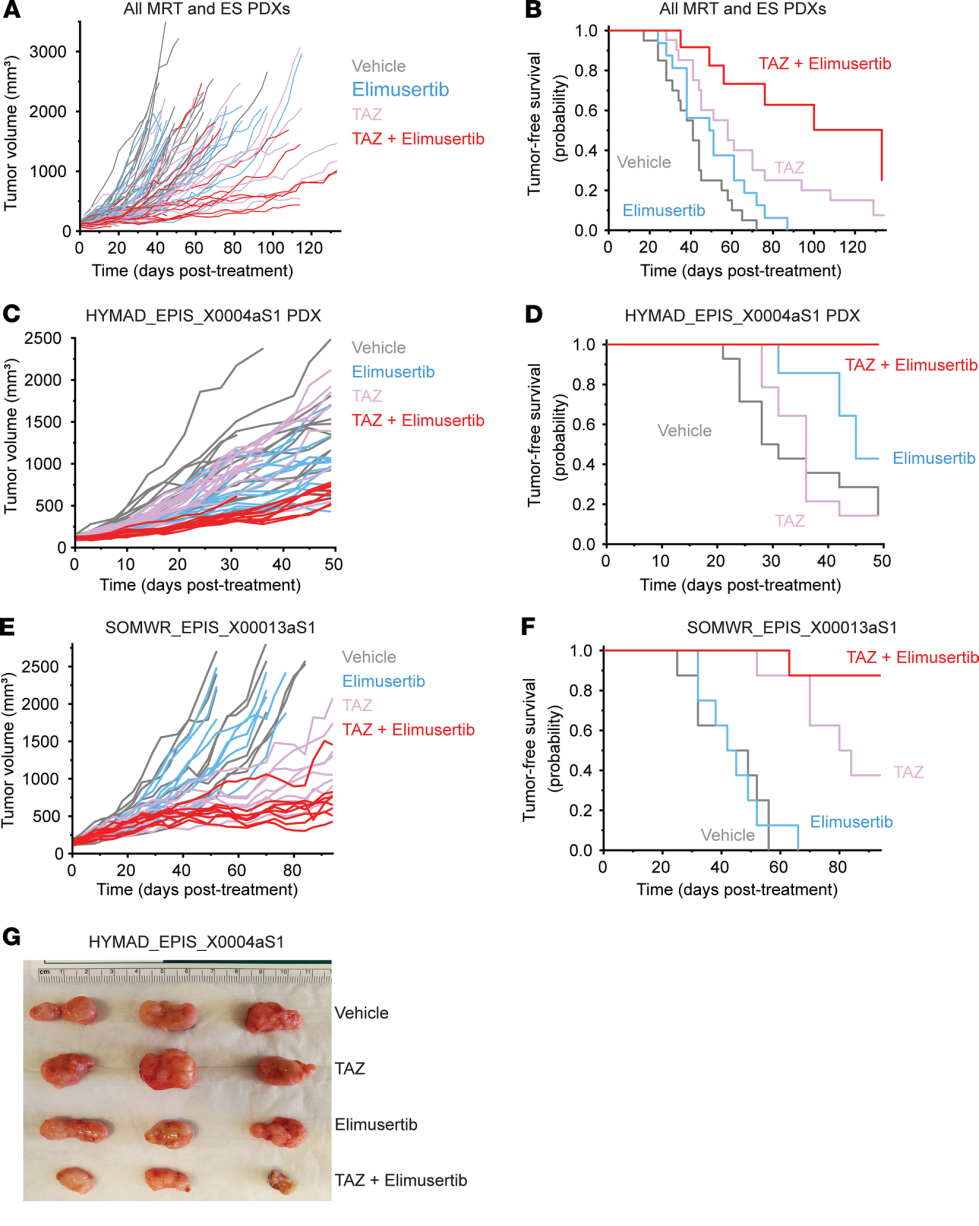
Tazemetostat plus elimusertib improves therapeutic response in vivo. (**A**) Tumor growth curves for 5 mouse PDXs treated with the indicated drug regimen. *n* = 20 mice for vehicle and elimusertib groups, *n* = 21 for TAZ and TAZ plus elimusertib groups. *P* = 0.023 and 0.20, Vardi *U* test, for combination versus elimusertib or TAZ, respectively. (**B**) Kaplan-Meier curves showing tumor-free survival (defined as tumor volume ≤1,000 mm^3^) for the PDXs in panel **C**. Mean survival is 51 days (95% CI 42–60) for elimusertib, 68 days (95% CI 53–82) for TAZ to 100 days (95% CI 74–124) for the combination. Log-rank test *P* = 5.8 × 10^–4^ and 0.038 for combination versus elimusertib or TAZ, respectively. (**C**) Tumor growth curves for the HYMAD_EPIS_X0004aS1 PDX model treated with the indicated drug regimen. *P* = 2.0 × 10^–4^, Vardi *U* test, for combination versus elimusertib or TAZ. *n* = 14 mice per treatment group. (**D**) Kaplan-Meier curves showing tumor-free survival for the PDXs in panel **C**. Log-rank test *P* = 0.0062 and 6.3 × 10^–5^ for combination versus elimusertib or TAZ, respectively. (**E**) Tumor growth curves for the SOMWR_EPIS_X00013aS1 PDX model. *P* = 0.001 and 0.06, Vardi *U* test, for combination versus elimusertib and TAZ, respectively. *n* = 8 mice for all groups. (**F**) Kaplan-Meier curves showing tumor-free survival for the SOMWR_EPIS_X00013aS1 PDX model; mean survival is 90 days (95% CI 83–97) for combination versus 80 days (95% CI 70–90) for TAZ and 45 days (95% CI 37–52) for elimusertib. Log-rank test *P* = 9.5 × 10^–5^ and 0.06 for combination versus elimusertib and TAZ, respectively. (**G**) Image of representative tumors extracted from mice in **C** and **D** on day 52 of treatment.

This nominates therapeutic targeting of the EZH2-PGBD5 synthetic lethal dependency as an improved combination strategy for epithelioid and rhabdoid tumors. To test this idea, we assembled a phase 2–like cohort of diverse rhabdoid and epithelioid sarcoma tumors derived from patients with relapsed and metastatic disease, including tumors with numerous additional acquired mutations (Supplemental Table 4). We engrafted these tumors into immunodeficient *NOD-scid IL2Rgamma^null^* mice and randomized tumor-bearing animals to treatment with tazemetostat or elimusertib or the combination of both (Figure 5A). The combination of tazemetostat and elimusertib exceeded the effect of treatment with either drug alone when assessed by tumor growth measurements (*P* = 0.023 and 0.20, by Vardi *U* test, for combination versus elimusertib or tazemetostat, respectively) (Figure 5A) and significantly extended tumor-free survival from 51 days (95% CI 42–60) for elimusertib and 68 days (95% CI 53–82) for tazemetostat to 100 days (95% CI 76–124) for the combination (*P* = 5.8 × 10^–4^ and 0.038, log-rank test, for combination versus elimusertib or tazemetostat, respectively) (Figure 5B).

This activity was most pronounced for the HYMAD_EPIS_X0004aS1 and SOMWR_EPIS_X00013aS1 patient-derived xenograft (PDX) models (Figure 5, C–G), despite the former exhibiting a relatively poor response to tazemetostat monotherapy, when assessed by tumor growth measurements (*P* = 2.0 × 10^–4^, by Vardi *U* test, for combination versus elimusertib or tazemetostat for HYMAD_EPIS_X0003aS1, Figure 5C; *P* = 0.001 and 0.06 for combination versus elimusertib and tazemetostat, respectively, for SOMWR_EPIS_X00013aS1, Figure 5E) and tumor-free survival (*P* = 0.0062 and 6.3 × 10^–5^, by log-rank test*,* for combination versus elimusertib or tazemetostat, respectively, for HYMAD_EPIS_X0003aS1, Figure 5D; and *P* = 9.5 × 10^–5^ and 0.06 for combination versus elimusertib or tazemetostat, respectively, for SOMWR_EPIS_X00013aS1, Figure 5F). This effect leverages the EZH2-PGBD5 collateral synthetic lethal dependency, targeting PGBD5-dependent DNA damage to improve tazemetostat clinical response and overcome resistance in epithelioid and rhabdoid tumors.

## Discussion

Prior studies showed that inhibition of EZH2 methyltransferase activity using tazemetostat is insufficient to induce durable tumor regressions in most patients with epithelioid and rhabdoid tumors (8). Our recent functional genetic studies of patients’ tumors before and after clinical tazemetostat therapy led to a specific model of effective epigenetic therapy, including rational combinations to overcome RB1/E2F pathway defects that were observed in 43% of tumors with primary or acquired tazemetostat resistance (8). Here, we advanced this approach further by identifying a collateral synthetic lethal dependency between EZH2 and PGBD5 in rhabdoid and epithelioid sarcomas that confers a susceptibility to combined epigenetic therapy using EZH2 and ATR inhibition. We found that EZH2 inhibition can upregulate transposase-derived PGBD5, which induces DNA damage, requiring ATR- but not CHK1-mediated DNA damage repair signaling in *SMARCB1*-deficient rhabdoid and epithelioid tumors regardless of their *RB1* mutational status. As a result, combined EZH2 and ATR inhibition exerts synergistic antitumor effects, as measured by DNA damage induction in vitro and tumor growth reduction and improvements in tumor-free survival in vivo.

The combination of EZH2 and ATR inhibition offers both a promising therapeutic approach that can be rapidly translated to clinical trials for patients with rhabdoid and epithelioid sarcomas and a compelling example of drug-induced synthetic lethality. Originally described as genetic interactions (21), synthetic lethal targeting has proven to be a powerful therapeutic approach for the treatment of cancers. For example, breast and ovarian carcinomas with *BRCA1/2* mutations exhibit increased dependence on poly(adenosine diphosphate-ribose) polymerase–mediated (PARP-mediated) DNA repair, conferring a susceptibility to PARP1/2 inhibitors such as olaparib (22, 23). Synthetic dependencies in DNA damage signaling, chromatin remodeling, and metabolic functions have recently been defined to develop improved targeted therapies (24–28). In particular, acquired defects in DNA repair in tumor cells, such as inactivating mutations or functional defects in ATM signaling, can confer susceptibility to ATR inhibition due to the specific requirements of concurrent ATM and ATR signaling for efficient end-joining DNA repair and tumor cell survival (28–32).

ATR-selective inhibitors also synergize with genotoxic chemotherapies, such as DNA cross-linking platinum drugs, due to the specific requirements of ATR-dependent DNA repair during DNA replication (28). However, this approach has limited therapeutic efficacy due to its effects on healthy cells. Thus, effective targeting of DNA damage repair must leverage tumor-specific mutational and repair processes, providing a rationale for combining epigenetic and DNA repair inhibitors. In the case of rhabdoid and epithelioid sarcomas with *SMARCB1* deficiency, the epigenetic antagonism between the chromatin-remodeling activities of BAF and PRC2 contributes to the dependency of tumor cells on EZH2 activity (5), whose inhibition promotes the expression of tumor suppressors otherwise repressed by PRC2 (8, 33). This epigenetic reprogramming also appears to increase the expression of PGBD5, with the associated DNA damage and requirement for its ATR-dependent repair. It remains to be defined whether this effect is due to direct repression through PRC2-mediated histone 3 lysine 27 trimethylation of the *PGBD5* locus and/or indirect transcriptional or post-transcriptional regulation of *PGBD5* expression.

Though the cell of origin of rhabdoid and epithelioid sarcomas is currently unknown, these tumors exhibit epigenetic and transcriptional features of neuronal and neural crest cells (34–36). EZH2 is known to regulate neuronal differentiation (37–40); therefore, it will also be important to determine whether EZH2 inhibition of other PGBD5-expressing tumors, such as neuroblastomas, medulloblastomas, and Ewing and other fusion sarcomas, all of which also share features of neuronal lineages, can also induce PGBD5-dependent DNA damage, thereby conferring a susceptibility to collateral synthetic lethal targeting with ATR inhibitors. Importantly, susceptibility to ATR inhibition can also result from PGBD5-independent sources of intrinsic DNA damage, such as alternative lengthening of telomeres (41), replication stress due to transcriptional-replication interference (42–44), and functional ATM defects (30, 45), all of which may occur concurrently in tumor cells undergoing PGBD5-dependent DNA damage. Similarly, EZH2 may also contribute to other mechanisms of DNA damage repair (46–53). Thus, future biochemical and genetic studies will be needed to define specific mechanisms of EZH2- and ATR-dependent DNA damage repair signaling in tumor and healthy cells to identify improved targets to develop exclusively tumor-selective therapies. This direction is particularly compelling because PGBD5-dependent DNA damage repair appears to require ATR but not CHK1 kinase signaling in epithelioid and rhabdoid tumor cells, in contrast to ATR/CHK1-dependent canonical signaling observed during replication stress in healthy tissues.

Further definition of the EZH2-PGBD5 collateral synthetic lethal mechanisms should also aid in therapy stratification of combined EZH2 and ATR inhibition. For example, apparent variation in response to the tazemetostat-elimusertib combination between patient-derived epithelioid and rhabdoid tumors may result from biological differences in DNA damage repair signaling, PGBD5 activity, or other sources of intrinsic DNA damage, which, in turn, may be associated with the recently described distinct molecular subtypes of rhabdoid and epithelioid sarcoma tumors (54). In addition, both EZH2 and ATR inhibition can be immunogenic (8, 55–57), and studies will be needed to define immunologic effects of this combination therapy that may contribute to therapeutic effects in patients. In all, this work emphasizes how improved understanding of collateral dependencies of intrinsic mutators and epigenetic dysregulation responsible for causing childhood and young-onset cancers can be leveraged for rational combination therapies.

## Methods

### Sex as a biological variable.

Our study examined male and female animals, and similar findings are reported for both sexes.

### Cell culture.

G401, TTC642, VAESBJ, TM8716, and RPE-hTERT cell lines were obtained from the American Type Culture Collection. The ES1 cell line was generated in house. Rhabdoid tumor cell lines KP-MRT-NS and KP-MRT-RY were provided by Yasumichi Kuwahara and Hajime Hosoi, Department of Biochemistry and Molecular Biology, Kyoto Prefectural University of Medicine, Kyoto). The identity of all cell lines was verified by short tandem repeat analysis. Absence of *Mycoplasma* contamination was determined at every plating using the MycoAlert kit (Lonza) according to manufacturer’s instructions. Cell lines were cultured in 5% CO_2_ in a humidified atmosphere in 37°C. All media were obtained from Corning and supplemented with 10% FBS, 1% l-glutamine, and 100 U/mL penicillin and 100 μg/mL streptomycin (Gibco). G401, ES1, VAESBJ, and RPE cells were cultured in DMEM. TTC642, TM8716, KP-MRT-NS, and KP-MRT-RY cells were cultured in RPMI medium. All experiments were performed using cell lines kept in culture for no more than 10 passages. Generation of G401 with *RB1* deletion was described previously (8); the guide RNA sequence used is presented in Table 1.

### Western blotting.

To assess protein expression by Western immunoblotting, pellets of 1 million cells were prepared and washed once in cold PBS. Cells were resuspended in 100–130 μL of RIPA lysis buffer (50 mM Tris-HCl, pH 8.0, 150 mM NaCl, 1.0% NP-40, 0.5% sodium deoxycholate, 0.1% sodium dodecyl sulfate) and incubated on ice for 10 minutes. Cell suspensions were then disrupted using a Covaris S220 adaptive focused sonicator for 5 minutes (peak incident power: 35W; duty factor: 10%, 200 cycles/burst) at 4°C. Lysates were cleared by centrifugation at 18,000*g* for 15 minutes at 4°C. Protein concentration was assayed using the DC Protein Assay (Bio-Rad) and 15–35 μg whole-cell extract was used per sample. Samples were boiled for 5 minutes at 95°C in Laemmli buffer (Bio-Rad) with 40 mM DTT and resolved using SDS-PAGE. Proteins were transferred to Immobilon FL PVDF membranes (Millipore), and membranes were blocked using Intercept Blocking buffer (Li-Cor). Primary Abs (all from Cell Signaling Technology except where noted) used were as follows: anti-EZH2 (catalog 5246) at 1:1,000; anti-RPA32 pT21 (Abcam, ab109394) at 1:2,000; anti-RPA32 pS4/pS8 (Thermo Fisher Scientific, A300-245A) at 1:2,000; anti-RPA32 (catalog 35869) at 1:1,000; anti-pCHK1 S296 (catalog 90178) at 1:250, anti-MYCN (catalog 9405) at 1:250, and anti-actin (catalog 4970 and 3700) at 1:5,000. Blotted membranes were visualized using goat secondary Abs conjugated to IRDye 680RD or IRDye 800CW (Li-Cor, 926-68071 and 926-32210) at 1:15,000 and the Odyssey CLx fluorescence scanner, according to manufacturer’s instructions (Li-Cor). Image analysis was done using the Li-Cor Image Studio software (version 4).

### Transcriptomic data.

Expression levels of *PGBD5* and *MYCN* in G401 cells were determined from our previously published data set (8). Transcriptomic data (Figure 1, A and B) were generated and metaVIPER protein activity inference was performed as previously described (15, 16). Briefly, rhabdoid tumor transcriptomes referenced in this article were previously generated as part of the TARGET initiative (https://www.cancer.gov/ccg/research/genome-sequencing/target), with the dbGap study identification no. phs000218 and substudy identification no. phs000470 for all rhabdoid tumor samples. The data used for this analysis are available at Genomic Data Commons (https://portal.gdc.cancer.gov; TARGET-RT). Detailed methods for rhabdoid tumor sample processing may be found online (https://www.cancer.gov/ccg/research/genome-sequencing/target/using-target-data/technology#kidney-rhabdoid-tumor). Gene expression analysis and metaVIPER activity scores for each gene were previously generated by Coutinho et al. (16) and replotted in this work.

### Generating shPGBD5 cells.

For shRNA cells, pLKO.1 shRNA vectors targeting *PGBD5* (TRCN0000138412, TRCN0000135121) and control shGFP were obtained from the RNAi Consortium (Broad Institute). Lentivirus production was carried out as described previously (8). G401 cells were transduced at an MOI approximately 1.5 and selected with puromycin at 2 μg/mL for 72 hours. Knockdown was confirmed by quantitative RT-PCR as previously described (9), using primers specified in Table 1.

### Combination drug treatment.

Drugs used for in vitro treatment were supplied by Selleckchem (tazemetostat, S7128; elimusertib/BAY-1895344, S9864; camptothecin, S1288; SRA737, S8253).

For combination treatment with tazemetostat and elimusertib, we used a 2-dimensional dose-matrix design, treating the cells for 9 days. After the addition of cells, drugs were added using a pin tool (i.e., stainless steel pins with 50 nL slots; V&P Scientific) mounted onto a liquid-handling robot (CyBio Well vario; Analytik Jena). On day 9, CellTiter-Glo reagent was freshly reconstituted and added in a 1:1 proportion to cell medium, according to the manufacturer’s instructions. A similar protocol was used for monotherapy dose-response curves for elimusertib and SRA737, and for specific dose combinations, although drug solutions were added manually by multichannel pipette; specific treatment times are indicated in the corresponding figure legends. Outliers due to pinning errors were excluded after manual examination. For analysis of synergy, we used the SynergyFinder 3.0 website (58, 59), with contour plots based on calculated synergy generated in OriginPro (Microcal).

### Immunofluorescence.

Immunofluorescence for γH2AX was performed on cells plated on Millicell EZ Slide glass slides (EMD Millipore) coated for 45 minutes with bovine plasma fibronectin (Millipore Sigma). After drug treatment, cells were washed once with PBS and fixed in 4% formaldehyde for 10 minutes at room temperature. Slides were then washed 3 times in PBS for 5 minutes, permeabilized for 15 minutes in 0.3% Triton X-100, washed again in PBS 3 times, and blocked with 5% goat serum (Millipore Sigma, G9023) in PBS for 1 hour at room temperature. Slides were incubated with mouse anti-γH2A.X primary Ab (Sigma-Aldrich, 05-636) at 1:500 in blocking buffer for 1 hour, washed 3 times in PBS, and incubated with goat anti-mouse secondary Ab conjugated to AlexaFluor555 (Invitrogen, A-21422) at 1:1,000. Cells were then counterstained with DAPI at 1:1,000 for 10 minutes and treated with ProLong Diamond Antifade Mountant with DAPI (Invitrogen, P36962) for 48 hours.

Images were acquired on a Zeiss LSM880 confocal microscope at ×63 magnification. Images were then processed using a custom pipeline in CellProfiler (60). Per-cell integrated γH2A.X intensity was normalized against per-cell integrated DAPI intensity. All image analyses used the same pipeline settings, with the exception of the RescaleIntensity module for the AF555 channel, which used the settings 0.009–0.09 for the images in Figure 2 and 0.005–0.09 for Figure 4. Overlaid images were prepared using Fiji (61).

### Xenografts.

To generate PDXs, tumor specimens were immediately minced and mixed after collection (50:50) with Matrigel (Corning) and implanted subcutaneously in the flank of 6- to 8-week-old female NOD.Cg-*Prkdc^scid^ Il2rg^tm1Wjl^/Szj* (NSG) mice (The Jackson Laboratory), as described previously (62). Mice were monitored daily, and PDX samples were serially transplanted 3 times before being deemed established. PDX tumor histology was confirmed by review of H&E-stained slides and direct comparison to the corresponding patient tumor slides. PDX identity was further confirmed by Memorial Sloan Kettering Cancer Center–Integrated Mutation Profiling of Actionable Cancer Targets (MSK-IMPACT) sequencing analysis.

Therapeutic studies used female and male NSG mice obtained from The Jackson Laboratory. Xenografts were prepared as single-cell suspensions, resuspended in Matrigel, and implanted subcutaneously into the right flank of 6- to 10-week-old mice (100 μL of tumor cell suspension per mouse). Tumors were allowed to grow until they reached a volume of 100 μL, at which point they were randomized into treatment groups without blinding. Drugs were prepared using the following formulations: tazemetostat was dissolved at 25 mg/mL in 5% DMSO, 40% PEG 300, 5% Tween 80, and 50% water. Elimusertib was dissolved at 5 mg/mL in 10% DMSO, 40% PEG 300, 5% Tween 80, and 45% water using a sonicator. Drugs were reconstituted daily. tazemetostat was dosed at 250 mg/kg twice daily by oral gavage, 7 days/week. Elimusertib was dosed at 40 mg/kg twice daily by oral gavage using a 2-days-on/12-days-off cycle. Caliper tumor measurements were made twice weekly. Tumor volumes were calculated using the following formula: volume = (π/6) × length × width^2^.

### Statistics.

Data were analyzed using OriginPro (version 2022b; OriginLab Corporation), with the exception of tumor growth analysis, which was performed using the Vardi *U* test (63), as implemented in the *clinfun* R package using the aucVardiTest function. Tumor-free survival analysis was calculated by the Kaplan-Meier method, using the log-rank test. Data are presented as means, with individual replicates shown. Comparisons were performed by Student’s 2-tailed *t* test or permutation test with Benjamini-Hochberg correction for multiple comparisons as indicated. A *P* value <0.05 was considered significant.

### Study approvals.

All mouse experiments were carried out in accordance with Memorial Sloan Kettering Cancer Center’s IACUC-approved protocol 13-05-004 (8). Tumor specimens for PDX generation were collected under approved IRB protocol 14-091.

### Data availability.

All numerical data appearing in this article are included in the Supplemental Data Values file. Raw data and R scripts used for analysis of PDX data are available on Zenodo (DOI: 10.5281/zenodo.10398544). CellProfiler analysis pipeline files and raw image files are also available on Zenodo (DOI: 10.5281/zenodo.10982946).

## Author contributions

Y Kazansky and HSM designed and performed laboratory experiments, analyzed data, and drafted the manuscript. Y Kazansky and AK conceived of the project, and this was used to decide the order of coauthorship. DC, PD, and RQ performed laboratory experiments and analyzed data. ES oversaw PDX experiments. NZ, NA, VZ, Y Kuwahara, RS, FSDC, and ALK generated model systems used in this study. PSM and AC generated and analyzed metaVIPER data. NZ generated and provided the ES1 cell line. AK and MMG designed and oversaw the study and revised the manuscript. All authors reviewed and edited the manuscript.

## Supplementary Material

Unedited blot and gel images

Supplemental table 1

Supplemental table 2

Supplemental table 3

Supplemental table 4

Supporting data values

## Figures and Tables

**Table 1:**
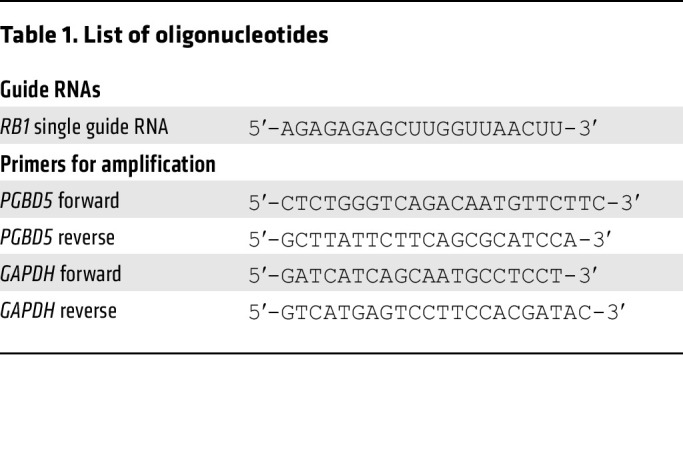
List of oligonucleotides

## Version 1 08/12/2025

In-Press Preview

## Version 2 10/15/2025

Electronic publication

## References

[1] Kadoch C, Crabtree GR (2015). Mammalian SWI/SNF chromatin remodeling complexes and cancer: Mechanistic insights gained from human genomics. Sci Adv.

[2] Versteege I (1998). Truncating mutations of hSNF5/INI1 in aggressive paediatric cancer. Nature.

[3] Kim KH (2015). SWI/SNF-mutant cancers depend on catalytic and non-catalytic activity of EZH2. Nat Med.

[4] McKenna ES (2008). Loss of the epigenetic tumor suppressor SNF5 leads to cancer without genomic instability. Mol Cell Biol.

[5] Wilson BG (2010). Epigenetic antagonism between polycomb and SWI/SNF complexes during oncogenic transformation. Cancer Cell.

[6] Kadoch C (2017). Dynamics of BAF-Polycomb complex opposition on heterochromatin in normal and oncogenic states. Nat Genet.

[7] Gounder M (2020). Tazemetostat in advanced epithelioid sarcoma with loss of INI1/SMARCB1: an international, open-label, phase 2 basket study. Lancet Oncol.

[8] Kazansky Y (2024). Overcoming clinical resistance to EZH2 inhibition using rational epigenetic combination therapy. Cancer Discov.

[9] Henssen AG (2017). PGBD5 promotes site-specific oncogenic mutations in human tumors. Nat Genet.

[10] Biegel JA (1999). Germ-line and acquired mutations of INI1 in atypical teratoid and rhabdoid tumors. Cancer Res.

[11] Chun HE (2019). Identification and analyses of extra-cranial and cranial rhabdoid tumor molecular subgroups reveal tumors with cytotoxic T cell infiltration. Cell Rep.

[12] Henssen AG (2015). Genomic DNA transposition induced by human PGBD5. Elife.

[13] Yamada M (2024). Childhood cancer mutagenesis caused by transposase-derived PGBD5. Sci Adv.

[14] Henssen AG (2017). Therapeutic targeting of PGBD5-induced DNA repair dependency in pediatric solid tumors. Sci Transl Med.

[15] Ding H (2018). Quantitative assessment of protein activity in orphan tissues and single cells using the metaVIPER algorithm. Nat Commun.

[16] Coutinho DF (2022). Validation of a non-oncogene encoded vulnerability to exportin 1 inhibition in pediatric renal tumors. Med.

[17] Mundi PS (2023). A transcriptome-based precision oncology platform for patient-therapy alignment in a diverse set of treatment-resistant malignancies. Cancer Discov.

[18] Lee CY (2024). Illuminating phenotypic drug responses of sarcoma cells to kinase inhibitors by phosphoproteomics. Mol Syst Biol.

[19] León TE (2020). *EZH2*-deficient T-cell acute lymphoblastic leukemia is sensitized to CHK1 inhibition through enhanced replication stress. Cancer Discov.

[20] Ngoi NYL (2024). Targeting ATR in patients with cancer. Nat Rev Clin Oncol.

[21] Dobzhansky T (1946). Genetics of natural populations; recombination and variability in populations of Drosophila pseudoobscura. Genetics.

[22] Farmer H (2005). Targeting the DNA repair defect in BRCA mutant cells as a therapeutic strategy. Nature.

[23] Bryant HE (2005). Specific killing of BRCA2-deficient tumours with inhibitors of poly(ADP-ribose) polymerase. Nature.

[24] Muller FL (2012). Passenger deletions generate therapeutic vulnerabilities in cancer. Nature.

[25] Zhao D (2017). Synthetic essentiality of chromatin remodelling factor CHD1 in PTEN-deficient cancer. Nature.

[26] Kwok M (2015). Synthetic lethality in chronic lymphocytic leukaemia with DNA damage response defects by targeting the ATR pathway. Lancet.

[27] Toledo LI (2021). A cell-based screen identifies ATR inhibitors with synthetic lethal properties for cancer-associated mutations. Nat Struct Mol Biol.

[28] Reaper PM (2011). Selective killing of ATM- or p53-deficient cancer cells through inhibition of ATR. Nat Chem Biol.

[29] Middleton FK (2015). Common cancer-associated imbalances in the DNA damage response confer sensitivity to single agent ATR inhibition. Oncotarget.

[30] Shruti M (2023). FET fusion oncoproteins disrupt physiologic DNA repair networks and induce ATR synthetic lethality in cancer. Res Sq.

[31] Min A (2017). AZD6738, a novel oral inhibitor of ATR, induces synthetic lethality with ATM deficiency in gastric cancer cells. Mol Cancer Ther.

[32] Menezes DL (2015). A synthetic lethal screen reveals enhanced sensitivity to ATR inhibitor treatment in mantle cell lymphoma with ATM loss-of-function. Mol Cancer Res.

[33] Knutson SK (2013). Durable tumor regression in genetically altered malignant rhabdoid tumors by inhibition of methyltransferase EZH2. Proc Natl Acad Sci U S A.

[34] Gadd S (2010). Rhabdoid tumor: gene expression clues to pathogenesis and potential therapeutic targets. Lab Invest.

[35] Custers L (2021). Somatic mutations and single-cell transcriptomes reveal the root of malignant rhabdoid tumours. Nat Commun.

[36] Chun HE (2016). Genome-wide profiles of extra-cranial malignant rhabdoid tumors reveal heterogeneity and dysregulated developmental pathways. Cancer Cell.

[37] Pereira JD (2010). Ezh2, the histone methyltransferase of PRC2, regulates the balance between self-renewal and differentiation in the cerebral cortex. Proc Natl Acad Sci U S A.

[38] Zhang J (2014). Ezh2 regulates adult hippocampal neurogenesis and memory. J Neurosci.

[39] Di Meglio T (2013). Ezh2 orchestrates topographic migration and connectivity of mouse precerebellar neurons. Science.

[40] Zhang M (2023). Neuronal histone methyltransferase EZH2 regulates neuronal morphogenesis, synaptic plasticity, and cognitive behavior in mice. Neurosci Bull.

[41] Flynn RL (2015). Alternative lengthening of telomeres renders cancer cells hypersensitive to ATR inhibitors. Science.

[42] Dorado García H (2022). Therapeutic targeting of ATR in alveolar rhabdomyosarcoma. Nat Commun.

[43] Zeman MK, Cimprich KA (2014). Causes and consequences of replication stress. Nat Cell Biol.

[44] Schoppy DW (2012). Oncogenic stress sensitizes murine cancers to hypomorphic suppression of ATR. J Clin Invest.

[45] Kwok M (2016). ATR inhibition induces synthetic lethality and overcomes chemoresistance in TP53- or ATM-defective chronic lymphocytic leukemia cells. Blood.

[46] Karakashev S (2020). EZH2 inhibition sensitizes CARM1-high, homologous recombination proficient ovarian cancers to PARP inhibition. Cancer Cell.

[47] Zhang X (2022). Combined inhibition of PARP and EZH2 for cancer treatment: Current status, opportunities, and challenges. Front Pharmacol.

[48] Ratz L (2022). Combined inhibition of EZH2 and ATM is synthetic lethal in BRCA1-deficient breast cancer. Breast Cancer Res.

[49] Campbell S (2013). Polycomb repressive complex 2 contributes to DNA double-strand break repair. Cell Cycle.

[50] Chou DM (2010). A chromatin localization screen reveals poly (ADP ribose)-regulated recruitment of the repressive polycomb and NuRD complexes to sites of DNA damage. Proc Natl Acad Sci U S A.

[51] Ito T (2018). Regulation of cellular senescence by polycomb chromatin modifiers through distinct DNA damage- and histone methylation-dependent pathways. Cell Rep.

[52] Piunti A (2014). Polycomb proteins control proliferation and transformation independently of cell cycle checkpoints by regulating DNA replication. Nat Commun.

[53] Rondinelli B (2017). EZH2 promotes degradation of stalled replication forks by recruiting MUS81 through histone H3 trimethylation. Nat Cell Biol.

[54] Pinto EM (2018). Malignant rhabdoid tumors originating within and outside the central nervous system are clinically and molecularly heterogeneous. Acta Neuropathol.

[55] Burr ML (2019). An evolutionarily conserved function of polycomb silences the MHC Class I antigen presentation pathway and enables immune evasion in cancer. Cancer Cell.

[56] Mehdipour P (2020). Epigenetic therapy induces transcription of inverted SINEs and ADAR1 dependency. Nature.

[57] Ishak CA (2016). An RB-EZH2 complex mediates silencing of repetitive DNA sequences. Mol Cell.

[58] Ianevski A (2020). SynergyFinder 2.0: visual analytics of multi-drug combination synergies. Nucleic Acids Res.

[59] Ianevski A (2022). SynergyFinder 3.0: an interactive analysis and consensus interpretation of multi-drug synergies across multiple samples. Nucleic Acids Res.

[60] Stirling DR (2021). CellProfiler 4: improvements in speed, utility and usability. BMC Bioinformatics.

[61] Schindelin J (2012). Fiji: an open-source platform for biological-image analysis. Nat Methods.

[62] Mattar M (2018). Establishing and maintaining an extensive library of patient-derived xenograft models. Front Oncol.

[63] Vardi Y (2001). Two-sample tests for growth curves under dependent right censoring. Biometrika.

