# Supplementary figures and images for: Epigenetic targeting of PGBD5-dependent DNA damage in SMARCB1-deficient sarcomas
Source: J Clin Invest. 2025 Aug 12;135(20):e179282. doi: 10.1172/JCI179282 (PMC12520684; doi:10.1172/JCI179282)

Figure 3B

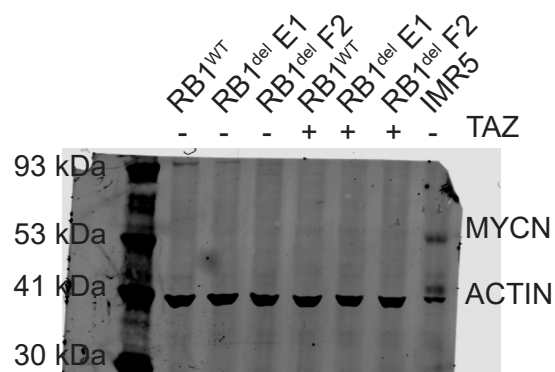

Figure 3C

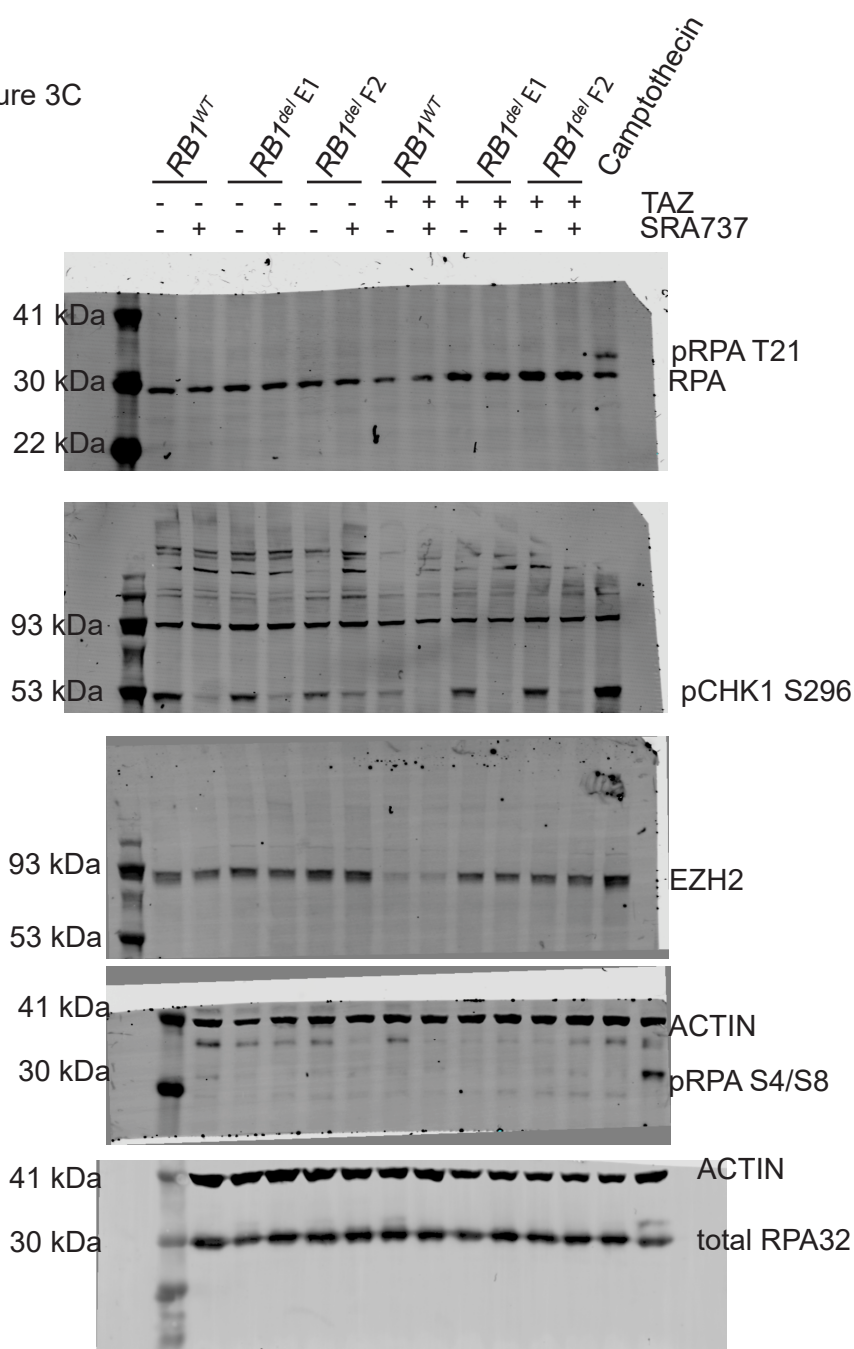

Supplement: Unedited blot and gel images [file jci-135-179282-s191.pdf]
